# On the Influence of Crosslinker on Template Complexation in Molecularly Imprinted Polymers: A Computational Study of Prepolymerization Mixture Events with Correlations to Template-Polymer Recognition Behavior and NMR Spectroscopic Studies

**DOI:** 10.3390/ijms150610622

**Published:** 2014-06-12

**Authors:** Siamak Shoravi, Gustaf D. Olsson, Björn C. G. Karlsson, Ian A. Nicholls

**Affiliations:** 1 Bioorganic & Biophysical Chemistry Laboratory, Linnæus University Centre for Biomaterials Chemistry, Linnæus University, SE-391 82 Kalmar, Sweden; E-Mails: siamak.shoravi@lnu.se (S.S.); gustaf.olsson@lnu.se (G.D.O.); bjorn.karlsson@lnu.se (B.C.G.K.); 2 Department of Chemistry—BMC, Uppsala University, P.O. Box 576, SE-751 23 Uppsala, Sweden

**Keywords:** molecular dynamics, molecular imprinting, molecularly imprinted polymer, nuclear magnetic resonance, propranolol

## Abstract

Aspects of the molecular-level basis for the function of ethylene glycol dimethacrylate and trimethylolproprane trimethacrylate crosslinked methacrylic acid copolymers molecularly imprinted with (*S*)-propranolol have been studied using a series of all-component and all-atom molecular dynamics studies of the corresponding prepolymerization systems. The crosslinking agents were observed to contribute to template complexation, and the results were contrasted with previously reported template-recognition behavior of the corresponding polymers. Differences in the extent to which the two crosslinkers interacted with the functional monomer were identified, and correlations were made to polymer-ligand recognition behavior and the results of nuclear magnetic resonance spectroscopic studies studies. This study demonstrates the importance of considering the functional monomer–crosslinker interaction when designing molecularly imprinted polymers, and highlights the often neglected general contribution of crosslinker to determining the nature of molecularly imprinted polymer-template selectivity.

## 1. Introduction

Materials capable of facilitating Angstrom- or nano-scale events such as in chemical catalysis or material-biomacromolecular interactions require architectures presenting functionalities and molecular-level features that permit high-fidelity molecular discrimination [1].

Molecular imprinting [2,3,4,5,6,7,8] is a technique for producing highly selective synthetic receptors for molecular structures spanning in size from ions to biomacromolecules. The method involves the formation of cavities in a synthetic polymer matrix that are of complementary structure and function to a template molecule/entity, a ligand for the synthetic receptor. The ability of molecularly imprinted polymers (MIPs) to selectively recognize and adsorb the imprinted ligand in the presence of closely related chemical species has led to the deployment of these materials in a range of biomedical and biotechnological applications, as antibody combining site or receptor binding site mimics. As such, MIPs have demonstrated affinities and cross-reactivity profiles comparable to their biological counterparts [9,10,11,12]. They have been employed as substitutes for biological antibodies in various application areas [13], e.g., medical diagnostic, forensic assay development, in solid phase extraction, as biosensor recognition elements and in catalysis. Recent examples of the use of MIPs in biological contexts [14,15,16] highlight the potential of molecular imprinting science and technology. The chemical and physical stabilities normally associated with molecularly imprinted materials [17] are factors further motivating the choice of MIPs as alternatives to biomacromolecules in many areas of application.

Historically, efforts to understand the molecular-level basis for the molecular imprinting process have focused on the interaction of functional monomers and template, as reflected in the development of a wide range of novel functional monomers [18,19,20,21], a focus that is also reflected in the early theoretical studies of MIP systems [22,23,24]. Throughout the development of the field, there has been a general awareness regarding the importance of crosslinking for capturing the functional monomer-template complexes and for determining polymer morphology. New crosslinking systems have been proposed in a number of studies; e.g., Kempe *et al.* explored a series of multi-acrylate crosslinking agents [25], Spivak *et al.* [26,27] presented functional monomers incorporating a second polymerizable moiety (conversely, crosslinking agents adorned with functionalities often associated with functional monomers) and Takeuchi explored the use of disulfide bonds in manipulating ligand recognition [28]. In more recent work, Piletsky *et al.* [29] have examined the possibility of orthogonal polymerization strategies where crosslinking of a monomer is accessible by more than one approach, Golker *et al.* [30] have studied the relationships between MIP-template recognition and template-crosslinker-functional monomer stoichiometries, and Henschel *et al.* [31,32] have explored the role of crosslinking monomer on MIPs developed for catalysis of the Diels-Alder reaction. Despite the acknowledgement of crosslinking as an important feature, little attention has been directed towards how the template-crosslinker interaction contributes to the recognition properties of the final material.

**Figure 1:**
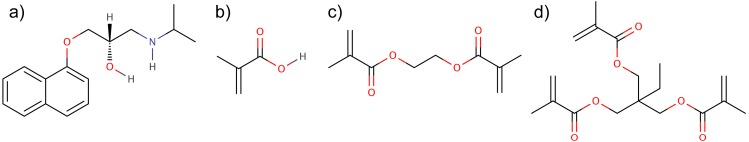
Structures of compounds used in this study: (**a**) the template (*S*)-propranolol (SPR); (**b**) the functional monomer methacrylic acid (MAA); (**c**) the crosslinking monomer ethylene glycol dimethacrylate (EGDMA) and (**d**) the crosslinking monomer trimethylolproprane trimethacrylate (TRIM).

In a seminal study by Andersson [33], the development of MIPs selective for the β-adrenergic antagonist (*S*)-propranolol (SPR) was described. The impact on template recognition of the two acrylate-based, crosslinking agents ethylene glycol dimethacrylate (EGDMA) and trimethylolproprane trimethacrylate (TRIM) when used in copolymers with the functional monomer methacrylic acid (MAA) was examined, as shown in Figure 1. SPR-MIPs have been the subject of a significant number of papers on account of interest in new methods for determining the enantiomeric purity of this important pharmaceutical and even in the establishment of these materials for use in the detection and removal of this compound from drinking water [34,35,36,37,38,39,40,41,42,43,44,45,46,47]. The initial success achieved with SPR-MIPs [33], together with propranolol’s inherent chirality, a valuable property for the development of reference or control studies, as well as its availability in radiolabeled form, have led to it being used as a “work-horse” template for fundamental investigations of the molecularly imprinted process, and for testing the suitability of MIPs in new application areas.

The desire to improve the efficiency of MIP design and development has motivated a growing number of studies aimed at creating a more detailed molecular-level understanding of the molecular imprinting process [48,49,50]. The recent development of full-system, all-atom molecular dynamics (MD) studies of MIP prepolymerization systems [51] has provided researchers with the tools for examining previously unexplored aspects of the molecular imprinting process [12,30,52,53,54,55,56]. The importance of the template-crosslinker interaction for MIP-template recognition has been an important observation in some of the systems studied, motivating further study of this aspect of the molecular imprinting process. In the present study, we have employed these computational tools to investigate the roles of EGDMA and TRIM in the SPR-MIP prepolymerization mixtures, and have drawn correlations with template recognition in the corresponding polymers, and with data from ^1^H-nuclear magnetic resonance (NMR) studies of the corresponding prepolymerization mixtures.

## 2. Results and Discussion

### 2.1. Molecular Dynamics Simulations

We elected to use full-system all-atom MD-based studies of two polymer systems previously described by Andersson [33] (polymers A and C in the original paper) to examine the molecular basis for template recognition in the EGDMA- and TRIM-crosslinked MAA copolymers reported in that seminal study. These polymers were prepared using the same solvent, initiator and the same template-functional monomer stoichiometry, though with different relative amounts of crosslinker. The MD simulations were performed using the same prepolymerization mixture stoichiometries as employed in the earlier studies (Table 1). Each system was simulated in quintuplet and interactions between species were evaluated using hydrogen bond analyses (Table 2 and Table 3). The hydrogen bond analyses revealed notable differences between the ensembles of interactions present in the two series of prepolymerization mixtures. This is reflected in the relative prevalence of interactions in the prepolymerization mixtures (here expressed as percentage of the simulated time) and is summarized in Table 2.

**Table 1:** Compositions of systems simulated (numbers of molecules present in mixture) ^a^.

Component	System A	System B
(*S*)-propranolol (SPR)	10	10
Ethylene glycol dimethacrylate (EGDMA)	398	-
Trimethylolproprane trimethacrylate (TRIM)	-	80
Methacrylic acid (MAA)	80	80
Toluene	1012	393
Azobisisobutyronitrile	6	2

^a^, System stoichiometries are representative of those of the polymers presented by Andersson [33].

In the case of the EGDMA copolymers, the crosslinker was observed to be the species present in the prepolymerization mixture that contributed most to the complexation of the template (System A, Table 2 and Table 3). On average, the template is engaged in interactions with the crosslinking monomer for roughly 55% of the simulation. This is a phenomenon similar to that observed in MD-studies of other EGDMA-MAA copolymers [30,51,52]. The predominance of EGDMA-template interactions was in stark contrast to the situation of the functional monomer MAA, which contributes relatively little to the template complexation (≈9% of time), though has a significant degree of association with EGDMA (≈62%). Closer analysis revealed that the acidic proton of MAA displays the most stable interactions observed (longest average lifetimes, Table 4), in particular with the carbonyl oxygen atoms of EGDMA, as well as the highest occupancy values observed in this system (Table 2 and Table 3).

**Table 2:** Time engaged in hydrogen bond formation (% of simulation) between species in Systems A and B.

Component	System A		System B	
	EGDMA	MAA	TRIM	MAA
SPR	55.5	8.5	35.8	30.8
MAA	61.9	*n.a.*	38.6	*n.a.*

*n.a.*, not analyzed.

**Table 3:** Observed average hydrogen bond occupancies ^a^.

Component	Atom	System A			System B			
		MAA	SPR		MAA	SPR		
		HAA	H28	H36	HAA	H28	H36	
SPR	N11	2.8 (2.23) ^b^	*n.a.*	*n.a.*	12.2 (2.20)	*n.a.*	*n.a.*	
	O6	0.1 (0.09)	*n.a.*	*n.a.*	0.2 (0.14)	*n.a.*	*n.a.*	
	O15	2.8 (1.63)	*n.a.*	*n.a.*	6.2 (1.74)	*n.a.*	*n.a.*	
MAA	OAC	*n.a.*^c^	0.1 (0.03)	0.2 (0.14)	*n.a.*	0.2 (0.11)	0.7 (0.31)	
	OAD	*n.a.*	1.0 (0.78)	1.6 (0.96)	*n.a.*	4.4 (1.20)	7.0 (0.85)	
EGDMA	O4	0.1 (0.02)	0.1 (0.07)	0.1 (0.05)	*-*	-	-	
	O7	0.1 (0.02)	0.1 (0.06)	0.0 (0.02)	-	-	-	
	O9	29.9 (2.67)	11.5 (1.65)	16.4 (4.11)	-	-	-	
	O13	31.8 (2.02)	10.3 (2.05)	17.0 (3.20)	-	-	-	
TRIM	O15	-	-	-	13.5 (1.15)	3.8 (1.40)	8.2 (3.39)	
	O22	-	-	-	12.3 (1.68)	3.8 (2.10)	8.1 (5.77)	
	O23	-	-	-	12.7 (1.82)	3.6 (1.38)	8.4 (3.41)	
	O4	-	-	-	0.0 (0.00)	0.0 (0.00)	0.0 (0.00)	
	O10	-	-	-	0.0 (0.00)	0.0 (0.00)	0.0 (0.00)	
	O17	-	-	-	0.0 (0.00)	0.0 (0.00)	0.0 (0.00)	

^a^, The values were calculated by summation of all observed occupancies (in percentage of simulation time) and for each analysed interaction and division of this sum by the number of reference molecules for each system. For all interactions involving the template (SPR) the occupancy values are calculated as “per template”. For all monomer-monomer contacts, the values were averaged against the number of functional monomers (MAA) being present at constant amounts in the evaluated systems. These values where then, again, summarized from each of the quintuplet simulations and a total average calculated from these results; ^b^, Values in brackets are the standard deviations for the average occupancies from quintuplet simulations; ^c^, *n.a.*, not analyzed.

When examining the TRIM-crosslinked polymers (System B), it was found that the extent of interaction of the crosslinker with the template was lower than in the EGDMA-system (Table 2 and Table 3). The extent of interaction of TRIM with the functional monomer was notably lower and in clear contrast to the situation in the EGDMA system. Further, the SPR-MAA interaction was considerably more prevalent in the TRIM system than in the case with EGDMA (≈31% compared with ≈9%, respectively). We also noted that from the perspective of the template, the total extent of interaction with monomers (crosslinker or MAA) over the 10 ns were quite similar, 64.0% and 66.6% for Systems A and B, respectively.

Initially, we attributed these differences in the extents of interactions to differences in the SPR:crosslinker and MAA:crosslinker stoichiometries employed in the original study, *i.e.*, 40 EGDMA molecules per template (System A) and 8 TRIM molecules per template (System B), corresponding to 80 carbonyl oxygens per template in System A and 24 carbonyl oxygen atoms per template in System B. Upon closer examination, we considered that the relative differences observed in the MD data (Table 2 and Table 3) could be dependent upon the number of crosslinker-derived hydrogen bond accepting atoms potentially available for interaction with the template, however the ≈3-fold lower total number of potential H-bond accepting carbonyl oxygens in the TRIM-system cannot alone account for the ≈0.3-fold lower degree of interaction of the template with this crosslinker, as compared to EGDMA. Furthermore, in the case of the crosslinker-MAA interactions, the extent of MAA’s interaction of MAA with the respective crosslinkers did not directly correlate with the differences in numbers of hydrogen bond acceptors available. Collectively, this suggested that despite the similarities in the functionalities present in these crosslinkers, there might be differences of a more fundamental character between these two cross-linking monomers when used in these two polymer systems.

**Table 4:** Observed average hydrogen bond lifetimes ^a^.

Component	Atom	System A			System C		
		MAA	SPR		MAA	SPR	
		HAA	H28	H36	HAA	H28	H36
SPR	N11	1.7 (0.30) ^b^	*n.a.*	*n.a.*	1.9 (0.04)	*n.a.*	*n.a.*
	O6	0.8 (0.13)	*n.a.*	*n.a.*	0.7 (0.03)	*n.a.*	*n.a.*
	O15	1.7 (0.13)	*n.a.*	*n.a.*	1.7 (0.14)	*n.a.*	*n.a.*
MAA	OAC	*n.a.* ^c^	0.6 (0.03)	0.7 (0.10)	*n.a.*	0.6 (0.02)	0.8 (0.06)
	OAD	*n.a.*	0.8 (0.05)	1.4 (0.47)	*n.a.*	0.8 (0.03)	1.6 (0.11)
EGDMA	O4	0.7 (0.03)	0.6 (0.06)	0.6 (0.03)	*-*	-	-
	O7	0.7 (0.03)	0.6 (0.05)	0.6 (0.05)	-	-	-
	O9	3.4 (0.09)	0.8 (0.05)	1.5 (0.15)	-	-	-
	O13	3.3 (0.13)	0.8 (0.04)	1.7 (0.10)	-	-	-
TRIM	O15	-	-	-	3.3 (0.06)	0.9 (0.06)	1.7 (0.26)
	O22	-	-	-	3.1 (0.20)	0.8 (0.03)	1.6 (0.26)
	O23	-	-	-	3.1 (0.15)	0.8 (0.04)	1.7 (0.26)
	O4	-	-	-	0.6 (0.03)	0.0 (0.00)	0.0 (0.00)
	O10	-	-	-	0.6 (0.03)	0.0 (0.00)	0.0 (0.00)
	O17	-	-	-	0.5 (0.03)	0.0 (0.00)	0.0 (0.00)

^a^, All time values are presented in picoseconds. The average interaction lifetimes were calculated by summation of all hydrogen bond event lifetimes and divided with the observed number of events in each quintuplet system. These average lifetimes were then summarized and averaged as the occupancies in Table 3. ^b^, Values in brackets are standard deviations of the average values presented in this table. ^c^, *n.a.*, not analyzed.

### 2.2. Correlations with Polymer-Template Binding and NMR Studies

To examine this further, comparisons with polymer-template recognition data were undertaken. In the original description of the binding of template by the EGDMA- and TRIM-based imprinted polymers [33], the IC_50_ values for binding in toluene were determined by radioligand binding studies to be 0.29 and 0.16 μM, respectively. Interestingly, the difference between these systems is reflected in the difference in the extent of MAA-SPR interactions observed in the MD studies of the prepolymerization mixtures of these polymers (stronger binding/lower IC_50_ and greater degree of MAA-template interaction). This suggested that the relatively limited MAA-TRIM interaction (in relation to the number of hydrogen-bond acceptors in each system) could provide a basis for the relative prevalence of the SPR-MAA interaction in System B. The basis for the lower extent of MAA-TRIM interaction per H-bond acceptor, as compared to the EGDMA system, was attributed to the comparatively greater steric crowding of hydrogen-bond acceptors of TRIM, relative to the situation in the EGDMA-based system. In other words, the third carbonyl of TRIM does not offer the same potential for interaction with the functional monomer or template as the first two, or those of EGDMA. This situation, in turn, leaves a greater proportion of the MAA available for relatively stronger interactions with the template, e.g., with the amine. While the degree to which the crosslinking afforded by the crosslinking monomers contributes to polymer-ligand recognition is not addressed here, it is the subject of ongoing theoretical and experimental studies in our laboratory. To the best of our knowledge, this is the first study in which the interplay between crosslinker and functional monomer can be seen to influence the degree of functional monomer–template interaction.

**Figure 2:**
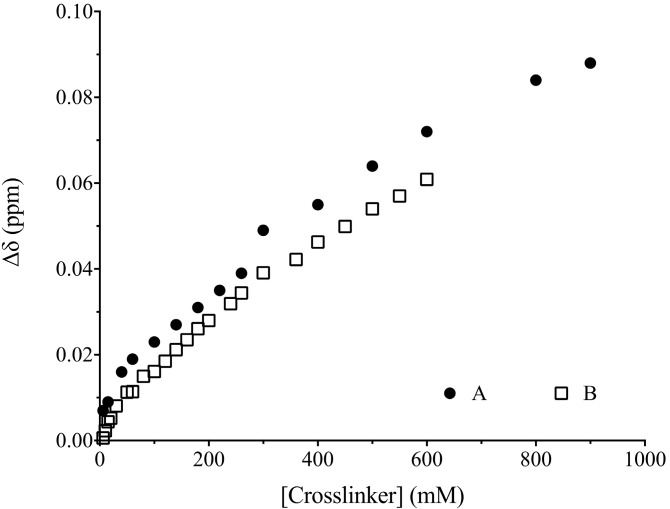
^1^H-NMR titration plots where constant concentrations of propranolol and methacrylic acid were titrated with increasing amounts of crosslinking monomer, in (A) TRIM and in (B) EGDMA. The isopropyl methyl resonances of (*S*)-propranolol were studied (Figure 1 and Figure 3).

To provide further insight, a series of ^1^H-NMR studies was undertaken where mixtures of SPR and MAA (the template and functional monomer had the same relative molar stoichiometries in both systems) were titrated with the crosslinkers, as shown in Figure 2. Using the methyl resonance of the isopropyl group of the template as a diagnostic tool and a one-site binding model, apparent *K*_d_ values of 696 ± 57 mM (standard error of the mean) and 641 ± 103 mM were determined for the crosslinker-SPR/MAA interaction in the EGDMA and TRIM systems, respectively. That the chemical shift changes of the template’s two equivalent methyl groups demonstrated no significant difference between the crosslinkers provides support for the interplay mechanism suggested by the MD-studies, whereby the competition of crosslinker and template for interaction with the functional monomer in both systems provides similar total degrees of complexation. Furthermore, the NMR data demonstrate the capacities of the two crosslinking agents to compete, albeit weakly, for coordination of the template in the presence of functional monomer. How the impact of crosslinker on the availability of functional monomer influences non-specific binding, and even how the degree of crosslinking may influence recognition are topics currently being addressed in our laboratory. This study also highlights the potential of functionalized cross-linking agents, such as those developed by Spivak *et al.* [26,27], for creating more homogeneous polymer recognition sites.

## 3. Experimental Section

### 3.1. Chemicals

(*S*)-propranolol hydrochloride, EGDMA, TRIM and *d*_8_-toluene were obtained from Sigma-Aldrich (Munich, Germany). Toluene was purchased from Merck (Solna, Sweden). MAA was distilled under reduced pressure and kept at −20 °C until used. (*S*)-propranolol was extracted from (*S*)-propranolol hydrochloride in a basic aqueous solution (pH ≈ 8) using diethyl ether as organic phase. EGDMA and TRIM were each extracted three times with a mixture of 75 mL 0.1 M NaOH and 25 mL saturated NaCl followed by 25 mL of saturated NaCl. The washed substances were dried over MgSO_4_ and passed through AlO_3_ prior to use.

### 3.2. Molecular Dynamics Simulations

All-atom MD simulations were conducted using the AMBER (v.10.0 UCSF, San Francisco, CA, USA) suite of programs [57,58] using a strategy previously described by Karlsson *et al.* [51] and developed in subsequent studies [30,52,55]. Implemented force field(s) in these simulations were the AMBER (FF03) [59] and the general amber force field (GAFF) [60]. Simulated systems were initially constructed using the PACKMOL software [61,62] to obtain random starting geometries, as presented by Olsson *et al.* [52] The compositions of the simulated all-component prepolymerization mixtures, as well as equilibration and production run data, are summarized in Table 1. All systems were simulated (in quintuplet) for 10 ns of recorded trajectory data.

**Figure 3:**
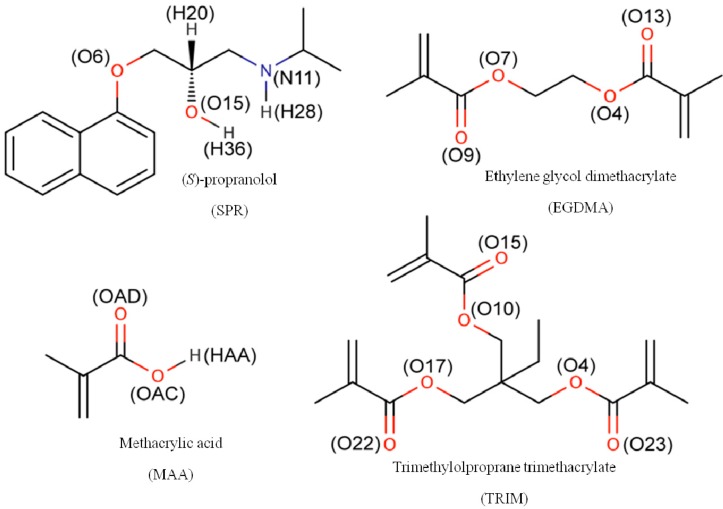
Structures of compounds used in this study with molecular abbreviations and atomic labels implemented in performed calculations and evaluations of modeled systems.

Production run trajectory datasets were analyzed using HBOND routine available in the PTRAJ module included in AmberTools (v. 1.3, UCSF, San Francisco, CA, USA) [57]. All hydrogen bond (HBOND) interactions were extracted from the trajectories using a distance and angle cut-off of 3.0 A and 60°, respectively. The structures of the analyzed molecular species and the interacting atoms potentially participating in hydrogen bond interactions are presented in Figure 3.

### 3.3. ^1^H-NMR Studies

All NMR studies were performed using *d*_8_-toluene as solvent on a Varian 500 MHz instrument (PaloAlto, CA, USA). Spectra were acquired at 293 ± 1 K using the Varian L700 Pulsed Gradient Driver steered with VNMR 6.1B software as supplied by the manufacturer.

Titration studies followed a previously described general protocol [63]: 17.7 mM (*S*)-propranolol solutions containing 142 mM MAA in *d*_8_-toluene were titrated using a 2 M EGDMA or TRIM solutions containing 17.7 mM propranolol and 142 mM MAA. Between 20 and 32 measurement points were used for each study.

## 4. Conclusions

Aspects of the molecular-level basis for the function of the crosslinking monomers EGDMA and TRIM in MAA copolymers imprinted with (*S*)-propranolol were studied using a series of all-component and all-atom MD simulations of the corresponding prepolymerization systems. The crosslinking agents were observed to make significant contributions to template complexation, though the MD studies reveal considerable differences in the balance of the ensembles of equilibria present in the polymerization systems. Our results indicate that differences in the extent that the crosslinkers interact with the functional monomer MAA contribute to its availability for interaction with the template, an effect not previously discussed in the literature. This study highlights the often neglected contribution of crosslinker in determining the nature of MIP-template selectivity. The results have correlated with the results of previously reported template-recognition studies of the corresponding polymers and with NMR studies of prepolymerization mixtures provided support for the conclusions drawn from the MD-studies. Finally, this study highlights the utility of all-atom full-system molecular dynamics studies as a diagnostic tool for the evaluation of molecular imprinting systems.

## References

[1] Zhang S.G. (2003). Fabrication of novel biomaterials through molecular self-assembly. Nat. Biotechnol..

[2] Sellergren B. (2001). Molecularly Imprinted Polymers: Man-Made Mimics of Antibodies and Their Applications in Analytical Chemistry.

[3] Alexander C., Andersson H.S., Andersson L.I., Ansell R.J., Kirsch N., Nicholls I.A., O’Mahony J., Whitcombe M.J. (2006). Molecular imprinting science and technology: A survey of the literature for the years up to and including 2003. J. Mol. Recognit..

[4] Whitcombe M.J., Kirsch N., Nicholls I.A. (2014). Molecular imprinting science and technology: A survey of the literature for the years 2004–2011. J. Mol. Recognit..

[5] Wulff G. (1995). Molecular imprinting in cross-linked materials with the aid of molecular templates—A way towards artificial antibodies. Angew. Chem. Int. Ed. Engl..

[6] Haupt K., Mosbach K. (2000). Molecularly imprinted polymers and their use in biomimetic sensors. Chem. Rev..

[7] Whitcombe M.J., Chianella I., Larcombe L., Piletsky S.A., Noble J., Porter R., Horgan A. (2011). The rational development of molecularly imprinted polymer-based sensors for protein detection. Chem. Soc. Rev..

[8] Komiyama M., Takeuchi T., Mukawa T., Asanuma H. (2004). Molecular Imprinting.

[9] Vlatakis G., Andersson L.I., Müller R., Mosbach K. (1993). Drug assay using antibody mimics made by molecular imprinting. Nature.

[10] Andersson L.I., Müller R., Vlatakis G., Mosbach K. (1995). Mimics of the binding sites of opioid receptors obtained by molecular imprinting of enkephalin and morphine. Proc. Natl. Acad. Sci. USA..

[11] Berglund J., Nicholls I.A., Lindbladh C., Mosbach K. (1996). Recognition in molecularly imprinted polymer α^2^-adrenoreceptor mimics. Bioorg. Med. Chem. Lett..

[12] Schillinger E., Möder M., Olsson G.D., Nicholls I.A., Sellergren B. (2012). An artificial estrogen receptor through combinatorial imprinting. Chem. Eur. J..

[13] Sellergren B., Allender C.J. (2005). Molecularly imprinted polymers: A bridge to advanced drug delivery. Adv. Drug Deliv. Rev..

[14] Hoshino Y., Koide H., Urakami T., Kanazawa H., Kodama T., Oku N., Shea K.J. (2010). Recognition, neutralization, and clearance of target peptides in the bloodstream of living mice by molecularly imprinted polymer nanoparticles: A plastic antibody. J. Am. Chem. Soc..

[15] Hoshino Y., Koide H., Furuya K., Haberaecker W.W., Lee S.-H., Kodama T., Kanazawa H., Oku N., Shea K.J. (2012). The rational design of a synthetic polymer nanoparticle that neutralizes a toxic peptide *in vivo*. Proc. Natl. Acad. Sci. USA.

[16] Piletska E.V., Stavroulakis G., Larcombe L.D., Whitcombe M.J., Sharma A., Primrose S., Robinson G.K., Piletsky S.A. (2011). Passive control of quorum sensing: Prevention of pseudomonas aeruginosa biofilm formation by imprinted polymers. Biomacromolecules.

[17] Svenson J., Nicholls I.A. (2001). On the thermal and chemical stability of molecularly imprinted polymers. Anal. Chim. Acta.

[18] Tanabe K., Takeuchi T., Matsui J., Ikebukuro K., Yano K., Karube I. (1995). Recognition of barbiturates in molecularly imprinted copolymers using multiple hydrogen-bonding. J. Chem. Soc. Chem. Commun..

[19] Piletsky S.A., Andersson H.S., Nicholls I.A. (1999). Combined hydrophobic and electrostatic interaction-based recognition in molecularly imprinted polymers. Macromolecules.

[20] Wulff G., Schonfeld R. (1998). Polymerizable amidines—Adhesion mediators and binding sites for molecular imprinting. Adv. Mater..

[21] Hall A.J., Manesiotis P., Emgenbroich M., Quaglia M., de Lorenzi E., Sellergren B. (2005). Urea host monomers for stoichiometric molecular imprinting of oxyanions. J. Org. Chem..

[22] Subrahmanyam S., Piletsky S.A., Piletska E.V., Chen B., Karim K., Turner A.P. (2001). “Bite-and-Switch” approach using computationally designed molecularly imprinted polymers for sensing of creatinine. Biosens. Bioelectron..

[23] Chianella I., Lotierzo M., Piletsky S.A., Tothill I.E., Chen B., Karim K., Turner A.P.F. (2002). Rational design of a polymer specific for microcystin-LR using a computational approach. Anal. Chem..

[24] Chianella I., Karim K., Piletska E.V., Preston C., Piletsky S.A. (2006). Computational design and synthesis of molecularly imprinted polymers with high binding capacity for pharmaceutical applications-model case: Adsorbent for abacavir. Anal. Chim. Acta.

[25] Kempe M. (1996). Antibody-mimicking polymers as chiral stationary phases in HPLC. Anal. Chem..

[26] Sibrian-Vazquez M., Spivak D.A. (2003). Enhanced enantioselectivity of molecularly imprinted polymers formulated with novel cross-linking monomers. Macromolecules.

[27] Sibrian-Vazquez M., Spivak D.A. (2004). Molecular imprinting made easy. J. Am. Chem. Soc..

[28] Takeda K., Kuwahara A., Ohmori K., Takeuchi T. (2009). Molecularly imprinted tunable binding sites based on conjugated prosthetic groups and ion-paired cofactors. J. Am. Chem. Soc..

[29] Lakshmi D., Whitcombe M.J., Davis F., Chianella I., Piletska E.V., Guerreiro A., Subrahmanyam S., Brito P.S., Fowler S.A., Piletsky S.A. (2009). Chimeric polymers formed from a monomer capable of free radical, oxidative and electrochemical polymerisation. Chem. Commun..

[30] Golker K., Karlsson B.C.G., Olsson G.D., Rosengren A.M., Nicholls I.A. (2013). Influence of composition and morphology on template recognition in molecularly imprinted polymers. Macromolecules.

[31] Kirsch N., Hedin-Dahlstrom J., Henschel H., Whitcombe M.J., Wikman S., Nicholls I.A. (2009). Molecularly imprinted polymer catalysis of a Diels-Alder reaction. J. Mol. Catal. B Enzym..

[32] Henschel H., Kirsch N., Hedin-Dahlstrom J., Whitcombe M.J., Wikman S., Nicholls I.A. (2011). Effect of the cross-linker on the general performance and temperature dependent behaviour of a molecularly imprinted polymer catalyst of a Diels-Alder reaction. J. Mol. Catal. B Enzym..

[33] Andersson L.I. (1996). Application of molecular imprinting to the development of aqueous buffer and organic solvent based radioligand binding assays for (*S*)-propranolol. Anal. Chem..

[34] Nilsson S., Schweitz L., Petersson M. (1997). Three approaches to enantiomer separation of β-adrenergic antagonists by capillary electrochromatography. Electrophoresis.

[35] Haginaka J., Sakai Y., Narimatsu S. (1998). Uniform-sized molecularly imprinted polymer material for propranolol recognition of propranolol and its metabolites. Anal. Sci..

[36] Mullett W.M., Martin P., Pawliszyn J. (2001). In-tube molecularly imprinted polymer solid-phase microextraction for the selective determination of propranolol. Anal. Chem..

[37] Schweitz L., Andersson L.I., Nilsson S. (2001). Rapid electrochromatographic enantiomer separations on short molecularly imprinted polymer monoliths. Anal. Chim. Acta.

[38] Fireman-Shoresh S., Popov I., Avnir D., Marx S. (2005). Enantioselective, chirally templated sol-gel thin films. J. Am. Chem. Soc..

[39] Schmidt R.H., Haupt K. (2005). Molecularly imprinted polymer films with binding properties enhanced by the reaction-induced phase separation of a sacrificial polymeric porogen. Chem. Mater..

[40] Schmidt R.H., Belmont A.S., Haupt K. (2005). Porogen formulations for obtaining molecularly imprinted polymers with optimized binding properties. Anal. Chim. Acta.

[41] Castell O.K., Allender C.J., Barrow D.A. (2006). Novel biphasic separations utilising highly selective molecularly imprinted polymers as biorecognition solvent extraction agents. Biosens. Bioelectron..

[42] Perez-Moral N., Mayes A.G. (2006). Direct rapid synthesis of MIP beads in SPE cartridges. Biosens. Bioelectron..

[43] Ye L., Yoshimatsu K., Kolodziej D., da Cruz Francisco J., Dey E.S. (2006). Preparation of molecularly imprinted polymers in supercritical carbon dioxide. J. Appl. Polym. Sci..

[44] Reimhult K., Yoshimatsu K., Risveden K., Chen S., Ye L., Krozer A. (2008). Characterization of QCM sensor surfaces coated with molecularly imprinted nanoparticles. Biosens. Bioelectron..

[45] Bompart M., Gheber L.A., de Wilde Y., Haupt K. (2009). Direct detection of analyte binding to single molecularly imprinted polymer particles by confocal Raman spectroscopy. Biosens. Bioelectron..

[46] Long Y., Philip J.Y.N., Schillen K., Liu F., Ye L. (2011). Insight into molecular imprinting in precipitation polymerization systems using solution NMR and dynamic light scattering. J. Mol. Recognit..

[47] Xu C., Ye L. (2011). Clickable molecularly imprinted nanoparticles. Chem. Commun..

[48] Nicholls I.A., Andersson H.S., Charlton C., Henschel H., Karlsson B.C.G., Karlsson J.G., O’Mahony J., Rosengren A.M., Rosengren J.K., Wikman S. (2009). Theoretical and computational strategies for rational molecularly imprinted polymer design. Biosens. Bioelectron..

[49] Nicholls I.A., Andersson H.S., Golker K., Henschel H., Karlsson B.C.G., Olsson G.D., Rosengren A.M., Shoravi S., Suriyanarayanan S., Wiklander J.G. (2011). Rational design of biomimetic molecularly imprinted materials: Theoretical and computational strategies for guiding nanoscale structured polymer development. Anal. Bioanal. Chem..

[50] Nicholls I.A., Karlsson B.C.G., Olsson G.D., Rosengren A.M. (2013). Computational strategies for the design and study of molecularly imprinted materials. Ind. Eng. Chem. Res..

[51] Karlsson B.C.G., O’Mahony J., Karlsson J.G., Bengtsson H., Eriksson L.A., Nicholls I.A. (2009). Structure and dynamics of monomer-template complexation: An explanation for molecularly imprinted polymer recognition site heterogeneity. J. Am. Chem. Soc..

[52] Olsson G.D., Karlsson B.C.G., Shoravi S., Wiklander J.G., Nicholls I.A. (2012). Mechanisms underlying molecularly imprinted polymer molecular memory and the role of crosslinker: Resolving debate on the nature of template recognition in phenylalanine anilide imprinted polymers. J. Mol. Recognit..

[53] Atta N.F., Hamed M.M., Abdel-Mageed A.M. (2010). Computational investigation and synthesis of a sol-gel imprinted material for sensing application of some biologically active molecules. Anal. Chim. Acta.

[54] Karlsson B.C.G., Rosengren A.M., Näslund I., Andersson P.O., Nicholls I.A. (2010). Synthetic human serum albumin sudlow I binding site mimics. J. Med. Chem..

[55] Olsson G.D., Karlsson B.C.G., Schillinger E., Sellergren B., Nicholls I.A. (2013). Theoretical studies of 17-β-estradiol-imprinted prepolymerization mixtures: Insights concerning the roles of cross-linking and functional monomers in template complexation and polymerization. Ind. Eng. Chem. Res..

[56] O’Mahony J., Karlsson B.C.G., Mizaikoff B., Nicholls I.A. (2007). Correlated theoretical, spectroscopic and X-ray crystallographic studies of a non-covalent molecularly imprinted polymerisation system. Analyst.

[57] Case D.A., Cheatham T.E., Darden T., Gohlke H., Luo R., Merz K.M., Onufriev A., Simmerling C., Wang B., Woods R.J. (2005). The Amber biomolecular simulation programs. J. Comput. Chem..

[58] Case D.A., Darden T.A., Cheatham T.E.I., Simmerling C.L., Wang J., Duke R.E., Luo R., Crowley M., Walker R.C., Zhang W. (2008). Amber 10.

[59] Duan Y., Wu C., Chowdhury S., Lee M.C., Xiong G., Zhang W., Yang R., Cieplak P., Luo R., Lee T. (2003). A point-charge force field for molecular mechanics simulations of proteins based on condensed-phase quantum mechanical calculations. J. Comput. Chem..

[60] Wang J., Wolf R.M., Caldwell J.W., Kollman P.A., Case D.A. (2004). Development and testing of a general amber force field. J. Comput. Chem..

[61] Martínez J.M., Martínez L. (2003). Packing optimization for automated generation of complex system’s initial configurations for molecular dynamics and docking. J. Comput. Chem..

[62] Martínez L., Andrade R., Birgin E.G., Martínez J.M. (2009). PACKMOL: A package for building initial configurations for molecular dynamics simulations. J. Comput. Chem..

[63] Svenson J., Andersson H.S., Piletsky S.A., Nicholls I.A. (1998). Spectroscopic studies of the molecular imprinting self-assembly process. J. Mol. Recognit..

